# Impact of lifestyle in prostate cancer patients. What should we do?

**DOI:** 10.1590/S1677-5538.IBJU.2021.0297

**Published:** 2021-08-15

**Authors:** Herney Andrés García-Perdomo, Juan Camilo Gómez-Ospina, María Juliana Chaves-Medina, Jesús Moreno Sierra, Ana María Autrán Gómez, Juan Gómez Rivas

**Affiliations:** 1 Universidad Del Valle School of Medicine Department of Surgery Cali Colombia Division of Urology/Uroooncology, Department of Surgery, School of Medicine, Universidad Del Valle, Cali, Colombia; 2 Universidad Del Valle School of Medicine UROGIV Research Group Cali Colombia UROGIV Research Group, School of Medicine, Universidad Del Valle, Cali, Colombia; 3 Hospital Clínico San Carlos Department of Urology Madrid Spain Department of Urology, Hospital Clínico San Carlos, Madrid, Spain; 4 Research Office of the Confederación Americana de Urología - CAU Madrid Spain Research Office of the Confederación Americana de Urología - CAU, Madrid, Spain

**Keywords:** Prostatic Neoplasms, Healthy Lifestyle, Diet

## Abstract

**Objective:**

This review aimed to analyze interventions raised within primary and tertiary prevention concerning the disease's incidence, progression, and recurrence of Prostate Cancer (PCa). Priority was given to the multidisciplinary approach of PCa patients with an emphasis on modifiable risk factors.

**Materials and Methods:**

We conducted a comprehensive literature review in the following databases: Embase, Central, and Medline. We included the most recent evidence assessing cohort studies, case-control studies, clinical trials, and systematic reviews published in the last five years. We only included studies in adults and in vitro or cell culture studies. The review was limited to English and Spanish articles.

**Results:**

Preventive interventions at all levels are the cornerstone of adherence to disease treatment and progression avoidance. The relationship in terms of healthy lifestyles is related to greater survival. The risk of developing cancer is associated to different eating habits, determined by geographic variations, possibly related to different genetic susceptibilities.

**Discussion:**

PCa is the second most common cancer in men, representing a leading cause of death among men in Latin America. Prevention strategies and healthy lifestyles are associated with higher survival rates in PCa patients. Also, screening for anxiety and the presence of symptoms related to mood disorders is essential in the patient's follow-up concerning their perception of the condition.

## INTRODUCTION

Cancer causes significant social and economic impacts, leading to 672.758 deaths per year worldwide. The increase in morbidity and mortality is directly related to the change in population distribution (1). Among the strongly linked factors, aging, increase in population life expectancy, better socioeconomic development worldwide, and the variable profile of pathologies stand out, placing cancer among the leading causes of death in the population (1).

Prostate cancer (PCa) is the second most common cancer in men, with a peak presentation between 65 and 70 years of age. It represents the leading cause of death among Latin American and Caribbean men (56.^4^ Age-Standardized Rate (ASR) per 100000) (2). It has a multifactorial and polygenetic etiology. Its incidence and prevalence are linked to modifiable and non-modifiable risk factors, which have been studied in different population groups. Non-modifiable factors include African descent and a family history of PCa; susceptibility is also described in individuals expressing certain genetic profiles. Among the modifiable factors, diet, exercise, toxic exposures, and behavioral measures have been linked to differences in PCa prognosis (3). In 2019, an estimate of 174.650 new PCa cases were diagnosed in the United States, representing one case for every five new cancer diagnoses, constituting a public health problem in the male population (1, 4). In Latin America, 190.385 new cases (27.9%) were reported (2). To reduce the social and economic impact on the population, guidelines have established primary, secondary, and tertiary prevention strategies (3, 5). In Latin America, prevention programs are heterogeneous due to the structure and accessibility of the different health systems.

This review focused on analyzing interventions raised within primary and tertiary prevention concerning the disease's incidence, progression, and recurrence of PCa. Priority was given to the multidisciplinary approach of PCa patients with an emphasis on modifiable risk factors.

### Acquisition of evidence

A comprehensive literature search was carried out in the following databases: Embase, Central, and Medline. We included studies that established the relationship between PCa and molecular factors, changes in diet (including dietary patterns, group relationship of macromolecules, vitamins, and micronutrients), exercise, mental health and work, and multimodal therapy; in terms of prevention, impact on morbidity-mortality and as part of treatment. We used MeSH terms related to PCa: “prostate cancer”, “prostate neoplasm” and for lifestyle both MeSH and free language terms were used for: exercise (“physical activity”, “aerobic exercise”, “exercise”, “training”); diet (“diet habit”, “diet modification”); mental health and work (“anxiety”, “mental health”, “depression”, “work”, “professional burnout”, “acute stress disorder”; and multimodal treatment (Supplementary material 1). We included the most recent evidence assessing cohort studies, case-control studies, clinical trials, and systematic reviews published in the last five years until September 2020. We only included studies in adults and in vitro or cell culture studies. The review was limited to English and Spanish articles. We included a few studies before five years, according to their relevance.

### Evidence synthesis

#### Molecular basis for the impact of lifestyle on PCa

We reviewed the molecular impact of lifestyle variation in subjects diagnosed with PCa. Those studies indicate that certain oncogenic factors are related to pro-inflammatory mechanisms, generating variations in the cellular cycle (Table-1). Among these factors are continuous exposure to androgens, insulin-like growth factor (IGF-1), vasoactive peptides, and reactive oxygen species (ROS) related to apoptosis inhibition. These enhanced abnormal cell signaling pathways overexpress angiogenesis mechanisms and cause disease progression (6, 7).

**Table 1:** Summary of the pathogenesis of molecular factors that are related to environmental factors such as diet and physical activity.

INTERVENTIONS	FACTORS	MOLECULE	MECHANISM OF ACTION		AUTHOR
**Physical activity**	Inflammation	IL2	↑	Innate immunity boosts	Thomas et al. 2017 (7)
		CRP	↑	Innate immunity boosts	
		IL6	↓	Inhibits caspases, antiproliferative	
		TNF	↓	Inhibits caspases, antiproliferative	
	Hormonal pathway modifiers	IGF-1	↓	Modification of hormonal pathways	
		IGFBP3	↑	Modification of hormonal pathways	
	Hormones	VIP	↓	Modifies androgenic resistance and cell proliferation	
		Irisin	↑	Modify bioavailability	
		Testosterone	↓ or ↑ or —	Modify bioavailability	
		Leptin	↓ or ↑ or —	Modifies cell proliferation	
	DNA	TMPRSS2: ERG	↓	Modify fusion	Pernar et al. 2019 (6)
		CRACR2A	Methylation	Modify expression	Dai et al. 2019 (8)
**Diet**	Carbohydrates	IGF-1 ↑	↑	Hyperinsulinemia, tumor development	Masko et al. 2013 (15)
	Proteins	Phytoestrogens	↓	Block in estrogen receptors	Hwang et al. 2009 (22)
		Heterocyclic amines	↑	Genomic instability	Koutros et al. 2008 (20)
	Fats	Omega 3	↓	Antiproliferative, antiangiogenic	Williams et al. 2011 (29)
	Vegetables	Isothiocyanates	↓	Block estrogen receptors	Gaziano et al. 2009 (36)
	Vitamins	Vitamin B	↓	Folate depletion	Tomaszewski et al. 2011 (35)
		Vitamin C	↓	Antioxidant	Gaziano et al. 2009 (36)
		Vitamin A	↑	Cell proliferation	Gaziano et al. 2009 (36)
	Phytochemicals	Epigallocatechin	↓	Antiproliferatives	Masko et al. 2013 (15)
		NF-kB	↓	Proapoptotic pathways	Masko et al. 2013 (15)
		Coffee	↓	Antioxidant	Xia et al. 2017 (42)
		Nuts	↓	Antioxidant	Pascual et al. 2018 (49)

* ↑: Increase activity; - no change ↓: Decrease activity IL: Interleukin; CRP: C-reactive protein; TNF: tumor necrosis factor; IGF-1: insulin-like growth factor-1; Insulin-like growth factor binding-protein-3 (IGFBP-3); VIP: Vasoactive intestinal peptide; TMPRSS2/ERG: gen fusion of Transmembrane protease serine 2 and ERG gen; CRACR2A: Ca^2+^ release-activated Ca^2+^ channel regulator 2A; NF-kB: nuclear factor kB.

DNA methylation, non-histone protein expression, and telomerase activity are modified under continuous exposure to ROS. Regular physical activity, and a diet low in carbohydrates and saturated fats are linked to suitable DNA structural repair. A prospective study has shown the relationship between vigorous physical activity and progression to metastatic PCa. Patients with regular physical activity one year before diagnosis showed decreased methylation of the promoter regions related to the CRACR2A gene. This gene encodes a calcium-binding protein, involved in the innate immune response, related with carcinogenesis mechanisms (8). Nevertheless, DNA structure hypomethylation is associated with a lower risk of progression. Thomas et al. found that sedentary patients, with high stress levels, overexpressed histone acetylation with excessive RAS oncogene family levels, which resulted in an increased cell proliferation. Also, microRNA (miRNA) generated cell signaling changes concerning the expression and coordination of p53 gene activity, concluding that physical activity is significantly related to epigenetic factors (7).

Qin et al. found that prostate cancer antigen 3 (PCA3) distinguishes healthy patients from patients with PCa; this biomarker is over-expressed in more than 95% of PCa cells. The influence of ethnicity on PCA3 is not clear; we need more studies to conclude differential diagnosis of PCa (9–11).

Patients with locally advanced PCa, in whom vigorous physical activity and absence of tobacco use was documented, had longer and more stable telomeres. Besides, they had a reduction in prostate-specific antigen (PSA) levels compared to sedentary patients. Increased expression of vasoactive intestinal peptide (VIP), which promotes angiogenesis, is related to rapid disease progression in patients with resistance to hormone therapy (8).

On the other hand, elevated testosterone levels are linked to an increase in PCa incidence. Physical activity, approximately 15 minutes to an hour, is known to increase testosterone levels quickly; however, increased levels are not related to free testosterone. They are directly related to higher protein-bound levels, which are biologically inactive. Over time, moderate to intense regular exercise reduces testosterone, generating negative feedback by blocking luteinizing and follicle stimulating hormones (7, 8).

Plant consumption was also related to increased expression of Interleukin 2 (IL-2). It promotes the innate immune response, generating adequate activity in natural killer (NK) and T cells. Patients with rich intake of cruciferous vegetables and isothiocyanates have a decreased expression of Interleukin 6 (IL-6) and Tumor Necrosis Factor (TNF), associated with the inhibition of caspase activation (7, 12).

At the molecular level, changes in diet and physical activity involving aerobic and anaerobic exercise on an ongoing basis (metabolic equivalents [MET] needed to perform the activity ≥6) are related to a modification in the hormonal and anti-inflammatory pathways. Regular physical activity alters endogenous hormone levels, reducing the availability of growth factors such as IGF-1, and decreasing cellular stress. No significant associations were found between total physical activity and PCa risk; however, men with vigorous physical activity continue to have a 30% lower risk of advanced PCa and a 25% lower risk of lethal PCa. The most common molecular subtype of PCa is related to the TMPRSS2:ERG gene fusion. Genetic expression of the TMPRSS2:ERG fusion gene is lower in men who perform vigorous physical activity compared to those who are sedentary (6).

On the other hand, sleep quality has been shown to have a buffering impact on cortisol levels. In a group of patients diagnosed with localized PCa, low cortisol levels were associated with depressive symptoms (13); also, low sleep quality was significantly associated to worse prognosis. These results suggest that the relationship between sleep quality and depressive symptoms may be partially explained by the altered circadian release of cortisol, influencing the inflammatory cascade that enhances disease progression and early mortality (13).

### Diet and PCa

Multiple studies have struggled to find the relationship between different dietary components and PCa at various disease stages. Also, different authors have searched the association between the impact of prevention strategies on PCa patients (Table-2).

**Table 2:** Summary with reviews that support intervention by food groups, discriminated by author, type of study, and intervention.

FOOD GROUP	AUTHOR	TYPE OF STUDY	FINDINGS
**Carbohydrates**	Fabiani et al. 2016 (16)	Systematic review and meta-analysis	Twelve observational studies were included. Patterns of "healthy diet" versus "Western diet" were compared in the risk of PCa. The Western diet increased the risk of PCa (OR 1.34). Also, a carbohydrate pattern was associated with increased risk (OR 1.64).
	Jayedi et al. 2018 (17)	Systematic review and meta-analysis	Twelve thousand four hundred ninety-four cases of PCa were included. The association between fasting glucose levels and the risk of PCa was determined. Increases in fasting glucose were not associated with an increased risk of PCa when comparing the category of high versus low levels (RR 0.88)
	Masko et al. 2013 (15)	Review	Studies of ketogenic non-carbohydrate diets showed a decrease in tumor growth in animal models. Low-carb diets (20%) showed similar tumor and survival growth rates. Human studies on the effect of restricting carbohydrates on PCa are lacking.
	Karlin et al. 2017 (18)	Case-control	They included 276 cases (patients with PCa and DM), 276 controls (patients with PCa without DM). Survival 5 years was 88% and 93% in patients with and without DM (HR 1.64). DM did not impact survival or PCa, and its treatment affected glycemic control.
**Proteins**	Cross et al. 2005 (19)	Prospective cohort	Cooking red meats can lead to the formation of heterocyclic amines, which are mutagenic compounds that are generated when creatine, amino acids, and sugars are subjected to high temperatures. These compounds can trigger genomic instability from direct DNA damage. More than 10 g a day of cooked meat than not consuming it was associated with an increased risk of PCa 1.4 times. One of the heterocyclic amines was associated with an increased risk of PCa of 1.2 times.
	Koutros et al. 2008 (20)	Prospective cohort	Six hundred sixty-eight patients with PCa (140 advanced) were included. Total meat consumption was associated with an increased risk of 1.26 times the incidence of PCa and 1.97 times the risk of advanced PCa when comparing the highest with the lowest consumption.
	Szymanski et al. 2010 (21)	Systematic review and meta-analysis	There was no association between fish consumption and reduced incidence of PCa. An association was found between fish consumption and a significant reduction of up to 63% in specific PCa mortality (RR 0.37).
	Hwang et al 2009 (22); Applegate et al 2018 (23)	Systematic review and meta-analysis	Soy contains high amounts of phytoestrogens, which can block the estrogen receptor, decrease proliferation, and increase cell differentiation, impacting PCa prevention. There is a significant association between soy consumption with a lower risk of PCa.
	Lu et al. 2016 (25)	Systematic review and meta-analysis	The partnership between dairy consumption and PCa mortality was assessed. An RR of 1.50 was found for whole milk. Increased whole milk consumption showed a high risk of prostate cancer mortality (RR1.43) in dose-response form.
**Fats**	Strom et al. 2008 (26)	Retrospective cohort	The association between saturated fat consumption and PCa biochemical relapse was assessed. Patients with high consumption had more relapse (p=0.006) and had shorter relapse-free survival than patients with low consumption of saturated fats (26.6 vs. 44.7 months, respectively, p=0.002).
	Gathirua-Mwangi and Zhang 2014 (27)	Systematic review	Studies have shown that consuming saturated fats has been significantly associated with an increased risk of advanced PCa.
	Berquin et al. 2011 (28)	Review	The increased proportion of unsaturated omega-6 fats versus omega-3s has been associated with an increased risk of PCa. Arachidonic acid is converted into other compounds that promote inflammation and cell growth. At the same time, omega-3 has anti-inflammatory, antiproliferative, antiangiogenic, and proapoptotic effects, which place it as an excellent anti-tumor molecule.
**Vegetables**	Liu et al. 2012 (33)	Systematic review and meta-analysis	The association between consumption of cruciferous vegetables, such as broccoli or cauliflower, and the risk of PCa was assessed. A significant decrease in the risk of PCa (relative risk 0.90) was found.
	Wang et al. 2006 (32)	In vitro	The effect of phenethyl isothiocyanate on the regulation of androgen receptor expression in PCa cells was studied. Cell growth was stopped, and androgen receptor expression decreased, mediating transcriptional and post-translational effects.
	Chan et al. 2009 (34)	Review	Vegetables of the genus allium, such as onion or garlic, can stimulate the immune system, inhibit cell growth, modulate the expression of androgen-responding genes and induce apoptosis so that they would play a protective role against PCa. However, their role in reducing the risk of PCa remains to be determined.
**Vitamins and minerals**	Masko et al. 2013 (15)	Review	Excessive vitamin A consumption has been associated with an increased risk of developing aggressive PCa, but not with the disease.
	Tomaszewski et al. 2011 (35)	In vitro	The level of serum folate and prostate tissue was evaluated and compared with cell proliferation. Serum folate levels correlated positively with folate content in tumor prostate tissue (p<0.03). Increased cell proliferation was found in patients with higher serum folate levels consistent with a higher incidence of PCa in patients receiving supplementation.
	Gaziano et al. 2009 (36)	Clinical trial	Compared with placebo, Vitamin E did not affect the incidence of PCa (HR 0.97; 0.58). Nor was a significant effect of vitamin C found in PCa (HR 1.02; px0.80).
	Gilbert et al. 2011 (37)	Systematic review and meta-analysis	In prospective studies, the OR for an increase of 1000 IU in daily intake was 1.14 for total PCa and 0.93 for aggressive PCa, and low vitamin D consumption could be associated with an increased risk of lethal PCa, no significant association with risk or its role in prevention or progression has been demonstrated.
	Klein et al. 2011 (38)	Clinical trial	The long-term effect of vitamin E supplementation on the risk of PCa was sought. The vitamin E group was at increased risk of developing PCa (HR 1.17; 0.008).
	Nimptsch et al. 2008 (39)	Prospective cohort	A non-significant inverse association between vitamin K consumption and PCa (relative risk 0.65) was observed. The association was stronger for the advanced PCa (relative risk 0.37, p for the trend 0.03).
	Stratton et al. 2011 (40)	Systematic review and meta-analysis	The use of multivitamin supplements, individual supplementation with vitamins or minerals did not affect PCa or the incidence of advanced or metastatic PCa or death by PCa.
**Phytochemicals**	Xia et al. 2017 (42)	Systematic review and meta-analysis	Consumption of coffee could reduce the risk of localized PCa (RR = 0.90, 95% CI: 0.84-0.97).
	Masko et al. 2013 (15)	Review	Epigallocatechin-gallate is the most abundant phytochemical of green tea. It has been described as reducing tumor growth in PCa, among other mechanisms such as induction of proapoptotic pathways, decreased inflammation through NF-kB, antioxidant properties. Its consumption could be associated with a lower incidence of PCa and a lower progression of precancerous lesions in dose-dependent form.
	Rowles et al. 2018 (45)	Systematic review and meta-analysis	A reduction in PCa incident risk was associated with high consumption of tomato and lycopene, a phytochemical of the carotenoid family, with antioxidant properties. The increase in tomato consumption was found to be inversely associated with the risk of PCa (RR 0.81, p=0.001), and a significant dose-response association was found.

### Carbohydrates

The role of simple carbohydrate consumption has been highlighted for its ability to generate hyperinsulinemia and obesity; likewise, insulin has been associated with tumor development. Previous studies related to ketogenic non-carbohydrate diets show decreased tumor growth in mice, similar to what is found in other low-carb diets, such as the Atkins diet (14, 15).

Fabiani et al. found that a carbohydrate diet pattern was associated with an increased risk of PCa (OR: 1.64) (16). However, the effects of IGF-1 have been controversial. According to Jayedi et al., increases in fasting blood glucose were not associated with an increased risk of PCa. Nonetheless, other studies show that the effect of blood glucose and diabetes on PCa is time-dependent. Thus, there could be a reduction of up to 12% in patients with type 2 diabetes mellitus (DM2). Consequently, similar survival may be seen in patients with or without DM2 (17, 18).

### Proteins

Proteins may be involved in tumor development or progression. Cooking red meats can lead to the formation of heterocyclic amines, which are mutagenic compounds that are generated when creatine, amino acids, and sugars are subjected to high temperatures. These compounds can trigger genomic instability from direct DNA damage, and a dose-response effect has been identified in PCa (19, 20). Other animal proteins, such as fatty fish, have not shown an association with an increased risk of PCa but could reduce the risk of cancer-specific death (21). Soy contains high amounts of phytoestrogens, which can block the estrogen receptor, decrease proliferation, and increase cell differentiation, which would contribute to PCa prevention, consistent with findings from a recent meta-analysis showing a significant association between soy consumption and a lower risk of PCa (22, 23).

The evidence is controversial, showing an association with PCa, but not with the aggressiveness or lethality (15). The relative risk (RR) of PCa associated with milk and dairy consumption has increased since the first studies, and the most recent meta-analysis reports an RR of 1.50 (IC95% 1.03-2.17) for whole milk. Thus, its use showed a high risk of prostate cancer mortality (RR 1.43 IC95% 1.13-1.81) (24, 25).

### Fats

So far, studies have found no association between overall fat consumption and PCa risk. However, consumption of saturated fats could be associated with an increased risk of biochemical recurrence following prostatectomy, along with an increased risk of advanced PCa (26, 27). On the other hand, the increased proportion of unsaturated Omega-6 fats versus Omega-3s has been associated with an increased risk of PCa. This is due to the conversion of arachidonic acid into other compounds that promote inflammation and cell growth (28). Omega-3, on the other hand, has anti-inflammatory, antiproliferative, antiangiogenic, and proapoptotic effects, which place it as an excellent anti-tumor molecule (29). Cholesterol can be a risk factor for the development of solid tumors due to pro-inflammatory pathway activation and intratumor steroidogenesis. In addition, low-density lipoproteins have been involved in the PCa onset and progression. Hence, some studies indicate that statins could prevent progression but not initiation of PCa (15, 30). Lippi et al. found a positive correlation between the intake of fried products and the risk of PCa, although the evidence was inconclusive (31).

### Vegetables

Epidemiological studies have found an inverse relationship between consumption of cruciferous vegetables (such as broccoli or cauliflower) and the risk of PCa. Isothiocyanates in this group of vegetables can suppress cell growth by inhibiting androgen receptor transcription (32, 33). Furthermore, vegetables of the genus Allium (e.g., onion, garlic) can stimulate the immune system, inhibit cell growth, modulate the expression of androgen-responding genes and induce apoptosis, playing a protective role against PCa (34).

### Vitamins and minerals

There is no clarity regarding vitamin B; however, evidence suggests that folate depletion could decrease tumor growth (35).

Similar effects have been attributed to vitamin C due to its antioxidant properties and its ability to reduce oxidative stress, which is an essential factor in cancer initiation and progression. However, randomized clinical trials have shown no effect on PCa (36).

Whereas low vitamin D consumption may be associated with an increased risk of PCa mortality, no significant association with regard to its role in prevention or progression has been demonstrated (37). Vitamin E supplementation was associated with an increased risk of cancer, although this was only evidenced in one study (38). By decreasing calcium bioavailability, vitamin K would expectedly be a protective factor in PCa, which was found in a cohort study (15, 39). Despite these studies, neither the use of multivitamin supplements nor individual vitamin or mineral supplementation affect the incidence of death from PCa (40).

### Phytochemicals

Epigallocatechin-gallate is the most abundant phytochemical of green tea, and its role in the reduction of PCa tumor growth has been reported. Other mechanisms, such as proapoptotic pathway induction, decreased inflammation through NF-kB, and antioxidant properties, have also been found. Its consumption could be associated with a lower incidence of PCa and a lower progression of precancerous lesions in a dose-dependent form (15).

Similarly, coffee has been inversely associated with PCa risk due to its caffeine content and other antioxidants (41). In the latest meta-analysis carried out by Xia et al., it was found that coffee consumption could reduce the risk of localized PCa (RR 0.90, IC95% 0.84-0.97) (42).

### Prevention of PCa

In general, measures are very similar to those taken to prevent cardiovascular risks, such as increasing consumption of fruits and vegetables, while reducing red meats and saturated fats. A reduction in PCa incidence risk has been associated with high tomato and lycopene consumption, a phytochemical of the carotenoid family, with antioxidant properties (43, 44). The most recent meta-analysis on tomato consumption and PCa risk found that the increase in consumption is inversely associated with the risk of PCa (RR 0.81, 0.71-0.92), and a dose-response association was found (45).

An increased risk of PCa with high consumption of beef, milk, or animal fat has been documented (5). More importantly, diet patterns seem to affect PCa progression. In this regard, people on predominantly Western diets (increased consumption of meats, processed products, and fats) have higher risk of PCa compared to those with high consumption of vegetables, fruits, and whole grains (16, 46). Meanwhile, the Mediterranean diet has encouraged the consumption of vegetables, fruits, nuts and seeds, whole grains, dairy products, olive oil, fresh fish, and seafood while restricting the intake of red and processed meats, fats, and sugary foods. A recent meta-analysis suggests that this dietary pattern is unrelated to overall PCa risk (RR 0.95 IC95% 0.90-1.01), or even cancer mortality (47). This diet and the so-called DASH have been inversely associated with the risk of aggressive PCa (48).

Pascual-Geller et al. found that a high intake of nuts and fish offered a protective function. In addition, there was a significant risk reduction of PCa in cases with higher consumption of fruits and vegetables, in addition to a lower risk of aggressiveness associated with eating fruits, vegetables, legumes, and fish. Similarly, Alvarez-Cubero et al. found that fruit consumption was associated with a lower Gleason score (49, 50). Notwithstanding, vegetarianism has not shown a significant reduction in the risk of PCa (51).

Based on the involvement of inflammation in the pathogenesis of some tumors such as PCa, the impact of some dietary components has been studied. Thus, saturated fats, refined sugars, and red meats could have pro-inflammatory properties, while some soy and phytochemical products would have anti-inflammatory properties. An increase in the diet's inflammatory index did not show an increase in the risk of PCa (RR 1.06 IC95% 0.97-1.15) (52). In contrast, antioxidant intake has been associated with a decrease in some biomarkers of oxidative stress in urine and benign prostate tissue in patients with PCa (53) (Supplementary material 2).

### Exercise and Pca

Studies on exercise and PCa have sought to establish their association with a decrease in tumor progression and mortality, as well as benefits in quality of life, post-prostatectomy urinary incontinence, and bone quality in patients with metastases, among others. Epidemiological studies have associated exercise and physical activity, including regular long-term recreational or occupational physical activity, with a decrease in cancer risk, specifically PCa, between 10 and 30%. Also, among the benefits of implementing an exercise regimen, there are: 1) Induction of epigenetic modifications; 2) Improvement in quality of life; 3) Regulation of the inflammatory response and immune system, and 5) Improvement in body composition by increasing muscle tissue (54–57).

There seems to be a significant benefit in decreasing exercise-related oxidative stress, which lowers hydrogen peroxide levels, a free radical involved in carcinogenesis, and other inflammatory mediators, including TNF-a and IL-6 (5, 7, 58). It is also important to note that a decreased risk is usually more significant with vigorous exercise than with light physical activity, demonstrating a dose-response effect (55). Multiple studies have shown a decrease in cancer-specific mortality associated with increased physical activity. On the other hand, Friedenreich et al. found a 38% reduction in PCa mortality in patients performing post-diagnostic physical activity (59). Also, prospective studies associate vigorous physical activity with decreased PCa mortality and reduced progression in patients with localized PCa (60).

Although physical activity has been shown to be of moderate benefit for fatigue reduction, sub-maximum physical condition, lower hemibody strength, perception of masculinity, body image, cognitive and social function, no significant effect has been found on the quality of life of PCa patients, disease progression, sexual function or cardiovascular health (61–63). In particular, endurance exercise improves muscle mass and strength in patients with PCa, without showing improvement in quality of life and fatigue. However, in patients treated with androgen deprivation therapy (ADT), there has been no difference between aerobic or resistance exercise (64, 65). It should be noted that leisure time physical activity, such as walking, swimming, or dancing, has not been associated with a lower risk of local or advanced PCa (66). Also, synergistically, exercise can lead to an increase in the potency of chemotherapy or radiation therapy by increasing circulation and intratumor chemotherapy delivery. Even as neoadjuvant therapy, exercise may be adequate in preparing and improving physical tolerance of patients who are taken to first-line chemotherapy (67). In patients taken to radical prostatectomy, pelvic floor exercises have shown to decrease the incidence of urinary incontinence significantly. However, these exercises do not appear to show any benefit when used only as a postoperative therapy (68).

Androgen deprivation therapy is generally considered a standard intervention in patients with locally advanced or metastatic PCa. However, it is associated with multiple adverse effects, such as reduction in muscle mass and strength, decrease in bone mass and density, in addition to increased fat mass, insulin resistance, fatigue, and alterations in sexual function (54). It has been shown that exercise can significantly improve cancer-related fatigue and quality of life in patients treated with ADT, as well as increase strength in the upper and lower body, help maintain control of fat mass and Body Mass Index (BMI), maintain sexual function, and mitigate resistance to some medications (such as enzalutamide). However, it has no substantial evidence for improvement of depression, bone mineral density or other blood markers (65, 67, 69, 70). Despite current evidence and recommendations on lifestyle interventions in PCa patients treated with ADT, patients continue to experience increased central adiposity, loss of bone density, and worsening of glycemia due to lack of adherence (71).

Men with more vigorous long-term physical activity had a 30% lower risk of progression to advanced PCa, and a 25% lower risk of dying from PCa (72). Also, muscle endurance exercises (non-aerobic) reduce the risk of tumor recurrence. It was also found that men with more robust physical activity had a lower risk of developing the TMPRSS2:ERG gene fusion (6).

### Mental health and PCa

Prostate cancer is a challenge for the mental health of patients even before they are diagnosed because of all the implications and interventions around it. During diagnostic procedures, specifically prostate biopsy, symptoms of depression or anxiety may be mild; however, when the biopsy is negative, anxiety symptoms are more significant in those with post-biopsy complications (pain, bruising, hematospermia, among other symptoms) (73). Screening with PSA has not been associated with increased concern or psychological distress. However, fear, anxiety, and lack of information can impact decisions and perceptions patients have about its performance (74, 75).

According to Watts et al., the prevalence of depression prior to, during, and after treatment for PCa was 17.27%, 14.7%, and 18.44%, respectively; while for anxiety, it was 27.04%, 15.09%, and 18.49%, respectively, both considerably high (76). Besides, mortality in patients with depression may be higher, and related emotional symptoms can negatively influence decision-making regarding therapy (77, 78).

Central concepts that influence the well-being of PCa patients include a sense of purpose, social connection, and motivation in life, which are suggested as essential areas of focus in practice (79). In this respect, patients with depressive symptoms benefit significantly from peer support or psychotherapy (80). Although no clinical trials were found to evaluate the efficacy of antidepressant medications in PCa patients, population studies reveal their regular use (81).

Patients with lower educational levels were found to be at increased risk of developing depressive symptoms, warranting relevant psychosocial interventions in high-risk groups (82). Furthermore, multidisciplinary interventions, such as online psychosocial support, can encourage that patients return to work, reducing the social and economic burden of PCa (83, 84).

Active surveillance has helped patients with low-risk PCa avoid or postpone active treatment and prevent possible adverse effects; however, the impact it may have on the psychosocial well-being of patients has not been well documented (85).

In support care, some unmet needs that merit person-centered care are identified. These include intimacy, information, physical, and psychological needs (86). Although radical prostatectomy, active surveillance, and radiation therapy are well tolerated in terms of post-treatment anxiety and depression, sexual and intestinal dysfunction, and, above all, urinary incontinence, can induce worsening of psychological distress (87).

Research regarding the impact of circadian cycle disruption on the risk of PCa, such as sleep disorders, or night shift work, which has inconsistently shown an increased risk of PCa predominantly in Asian populations, has been expanded in recent years. The risk is higher in rotating night shift work over fixed ones (88–91).

In addition to the adverse effects of ADT mentioned above, other psychological effects include, difficulties in multiple sexual domains, emotional lability, cognitive disturbances, reversible Parkinson's dementia, anxiety, and an increase in the risk for depression of up to 41%; these symptoms are significantly associated with alcohol consumption and smoking (92–95). Similarly, couples can also suffer emotional consequences related to the effects of ADT, with exercise being a useful intervention for the dyad (96). It has also been shown that the patient's partner may be afraid of recurrence of the disease, becoming a significant concern (97).

### Multimodal treatment and PCa

Having a pro-inflammatory cellular environment is one of the most important mechanisms in carcinogenesis. The presence of oxidative stress, inflammatory cytokines, and accelerated cell growth leads to a stable medium that predisposes mutations and expression of carcinogenic phenotypes (43, 98). Within the tertiary prevention of PCa, the MEAL study included 440 patients between the ages of 50 and 70, diagnosed with low-risk localized PCa (99). The progression of the disease was analyzed at the histological and biochemical level, in addition to the presence of urinary symptoms, a specific level of anxiety and quality of life (99); vegetables high in isothiocyanates, carotenes, and lycopene were found to be associated with PCa prevention (98, 99). Macro and micronutrient levels were measured in plasma during fasting, and results were generalizable to the general population; there were no significant differences between the age groups (p=0.98), race (p=0.52), geographic distribution (p=0.60), time of diagnosis of PCa (p=0.85), PSA values (p=0.96), clinical stage (p=0.27), and Gleason stratification (p=0.76) (99).

It was concluded that a healthy lifestyle, a diet low in saturated fats, and increased consumption of vegetables (more than six in a day) and legumes, were linked to stable or lower PSA levels, and a decrease in disease progression.

At the molecular level, changes in cellular expression, increased telomerase activity, and a higher stability in its terminals have been evidenced. The rates of intervention and progression of the disease were decreased, inhibiting carcinogenesis mechanisms and inducing the expression of cytoprotective enzymes (98, 99).

Patients with BMI >25 have increased levels of biomarkers linked to disease aggressiveness, such as C-peptide, insulin, insulin-like growth factor (IGF-1), IGF 3 binding protein, and adiponectin. They also have insulin receptor expression changes, IGF-1 receptor, and AKT receptor in epithelial and stromal cells, linked with rapid disease progression (70, 100). Free radical augmentation is generated from beta-oxidation during fat metabolism and induction of prostate inflammation, causing a significant increase in the risk of progression (101). Some authors propose inhibition of fatty acid synthase as a protective mechanism; however, documented studies are inconclusive (33, 42, 53, 71).

The active intervention of a plant-based diet, supplemented with soy, fish oil, vitamin E, selenium, and vitamin C, in conjunction with three hours per week of moderate exercise and one hour of daily stress management in support groups among patients with localized PCa, has shown slower carcinogenesis progression and decreased cell proliferation activity by approximately 70% (p <0.001). Satisfactory physical perception and lower levels of anxiety are reflected in quality of life assessment scales (102).

Dietary recommendations for patients with localized or metastatic castration resistant PCa have been the same as those that seek to reduce the risk of developing PCa (103). However, it has been considered whether some dietary products may have interactions with drugs used to treat castration resistant PCa. Pharmacokinetic studies have shown that the dosage of abiraterone with high or low-fat meals is associated with a modest increase in drug concentration compared to the usual fasting dosage (104). Szmulewitz et al. found that administering this drug with a low fat breakfast had a similar effect in terms of PSA levels and progression-free survival (105).

### Author's proposal

As noted above, the recommendations for lifestyle changes around PCa and its complications are not distant from the strategies for cardiovascular risk prevention. Currently, multiple molecular mechanisms appear to be involved in the development and progression of the disease, so even greater biological plausibility will be found to support these interventions.

As dietary measures, it is recommended to keep a low intake of simple carbohydrates, decrease consumption of red meats and processed foods, and reduce consumption of saturated fats. In addition, dietary patterns with high consumption of vegetables (predominantly cruciferous) and fruits, such as the Mediterranean diet, and high intake of tomatoes, may be suggested due to their lycopene content. Phytochemicals in tea and coffee are also beginning to play an essential role in the prevention of PCa and should be taken into account when guiding patients.

Muscular endurance exercise should be part of every doctor's general recommendations in the clinical field due to the potential benefit it confers in terms of cancer risk prevention and cardiovascular risk. These benefits are even more significant in patients diagnosed with PCa. Physical activity should be recommended throughout the disease process. It can improve patient's quality of life, urinary symptoms of incontinence, psychological stress resulting from the disease, and cancer-related fatigue until mortality is reduced; it also has the potential of being a significant neoadjuvant and adjuvant mode of therapy for the treatment of PCa. The introduction and promotion of multidisciplinary programs aimed at prevention, treatment, and palliation of PCa in Latin America are indispensable. A change in public policy is required to transform the reality of our patients.

These recommendations should be maintained for patients on ADT, in order to improve patient's quality of life. They may also be combating other mental health conditions, such as depression or anxiety, a widespread reality in these patients.

Priority is made to provide a comprehensive approach to PCa patients to influence all these factors and reduce the risk of progression. In this regard, the presence of anxious or depressive symptoms, tobacco use, or alcoholism should always be ruled out and alert to the presence of adverse psychosocial conditions that may worsen the prognosis and course of the disease. Psychosocial support should be included as part of standard management.

Establishing an adequate perception of the disease, while promoting protective factors, and educating patients on healthy lifestyles positively impacts the health system because of the decreased economic impact and greater adherence to treatment. Patients undergoing surgery, hormone therapy, and radiation therapy with joint monitoring of their lifestyle and risk factors may lower health costs and minimize the morbidity related to treatment (5, 55, 70, 99, 106).

**Figure 1:**
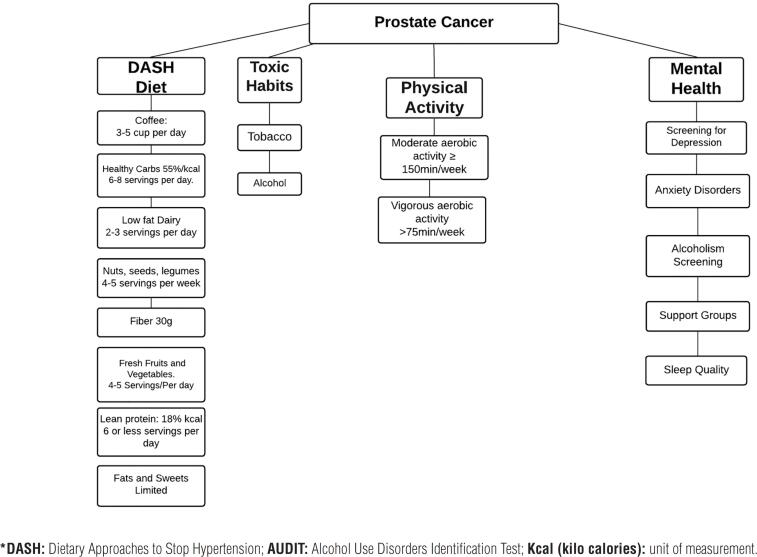
Summary outline of recommendations in healthy lifestyle.

Figure 1 and Supplementary material 2 summarize the recommendations for intervening in the lifestyle of patients.

## CONCLUSIONS

Preventive interventions at all levels are the cornerstone of adherence to disease treatment and progression avoidance. The relationship in terms of healthy lifestyles is related to greater survival. The risk of developing cancer is associated to different eating habits, determined by geographic variations, possibly related to different genetic susceptibilities.

Screening for anxiety and the presence of symptoms related to mood disorders is essential in the patient's follow-up concerning their perception of the condition.

## APPENDIX

**Supplementary material 1:** Search strategies. We used MeSH terms and synonyms related to prostate cancer and the suggested interventions.

**Diet and prostate cancer**
((((diet habit[MeSH Terms]) OR (diet habits[MeSH Terms])) OR (diet modification[MeSH Terms])) AND ((cancer of prostate[MeSH Terms]) OR (cancer of the prostate[MeSH Terms]) OR (cancer, prostate[MeSH Terms]) OR (prostate neoplasm[MeSH Terms])))
**Exercise**
(((activity, physical[MeSH Terms]) OR (aerobic exercise[MeSH Terms]) OR (exercise[title]) OR (physical activity[title]) OR (training[title])) AND ((cancer of prostate[MeSH Terms]) OR (cancer of the prostate[MeSH Terms]) OR (cancer, prostate[MeSH Terms]) OR (prostate neoplasm[MeSH Terms])))
**Mental health**
(((((anxiety[MeSH Terms]) OR (mental health[MeSH Terms])) OR (depression[MeSH Terms])) OR (work [MeSH Terms])) OR (burnout, professional[MeSH Terms])) OR (acute stress disorder[MeSH Terms]) AND ((cancer of prostate[MeSH Terms]) OR (cancer of the prostate[MeSH Terms]) OR (cancer, prostate[MeSH Terms]) OR (prostate neoplasm[MeSH Terms]))

**Supplementary material 2:** Summary of recommendations on lifestyle changes and Pca

**Diet** Consumption of cruciferous vegetables (broccoli, cauliflower) Increased consumption of tomato and its preparations Mediterranean diet pattern or DASH diet* Reduce consumption of simple carbohydrates and saturated fats Consumption of green tea and coffee (3-5 cups per day) Reduce consumption of red meats and processed products
**Exercise** At least 150 minutes/week Long-term physical activity Pelvic floor exercise for symptoms of urinary incontinence in post-prostatectomy patients Neoadjuvant or adjuvant exercise in patients on chemotherapy or radiation therapy, and in patients in ADT**
**Mental Health and Work** Screening for depressive, anxious, psychological stress symptoms Include psychosocial support within standard management Provide the opportunity for psychotherapy or peer support Comprehensive approach to the couple Avoid night shift jobs Providing multidisciplinary care to promote return to work
**Multimodal treatment** Close monitoring of all patients undergoing surgery, hormone therapy and radiation therapy with joint monitoring of their lifestyle and risk factors Similar recommendations in patients with castration-resistant metastatic PCa***
